# Metagenomic Insight into The Global Dissemination of The Antibiotic Resistome

**DOI:** 10.1002/advs.202303925

**Published:** 2023-10-23

**Authors:** Qi Zhang, Nuohan Xu, Chaotang Lei, Bingfeng Chen, Tingzhang Wang, Yunting Ma, Tao Lu, Josep Penuelas, Michael Gillings, Yong‐Guan Zhu, Zhengwei Fu, Haifeng Qian

**Affiliations:** ^1^ College of Environment Zhejiang University of Technology Hangzhou 310032 P. R. China; ^2^ Key Laboratory of Microbial Technology and Bioinformatics of Zhejiang Province Hangzhou 310012 P. R. China; ^3^ CSIC Global Ecology Unit CREAF‐CSIC‐UAB Bellaterra Barcelona Catalonia 08193 Spain; ^4^ CREAF Campus Universitat Autònoma de Barcelona Cerdanyola del Vallès Barcelona Catalonia 08193 Spain; ^5^ ARC Centre of Excellence in Synthetic Biology School of Natural Sciences Macquarie University Sydney NSW 2109 Australia; ^6^ Key Laboratory of Urban Environment and Health Institute of Urban Environment Chinese Academy of Sciences Xiamen 361021 P. R. China; ^7^ State Key Laboratory of Urban and Regional Ecology Research Center for Eco‐environmental Sciences Chinese Academy of Sciences Beijing 100085 P. R. China; ^8^ College of Biotechnology and Bioengineering Zhejiang University of Technology Hangzhou 310032 P. R. China

**Keywords:** antibiotic resistome, horizontal gene transfer, metadata, metagenome, one health

## Abstract

The global crisis in antimicrobial resistance continues to grow. Estimating the risks of antibiotic resistance transmission across habitats is hindered by the lack of data on mobility and habitat‐specificity. Metagenomic samples of 6092 are analyzed to delineate the unique core resistomes from human feces and seven other habitats. This is found that most resistance genes (≈85%) are transmitted between external habitats and human feces. This suggests that human feces are broadly representative of the global resistome and are potentially a hub for accumulating and disseminating resistance genes. The analysis found that resistance genes with ancient horizontal gene transfer (HGT) events have a higher efficiency of transfer across habitats, suggesting that HGT may be the main driver for forming unique but partly shared resistomes in all habitats. Importantly, the human fecal resistome is historically different and influenced by HGT and age. The most important routes of cross‐transmission of resistance are from the atmosphere, buildings, and animals to humans. These habitats should receive more attention for future prevention of antimicrobial resistance. The study will disentangle transmission routes of resistance genes between humans and other habitats in a One Health framework and can identify strategies for controlling the ongoing dissemination and antibiotic resistance.

## Graphical abstract

The global crisis in antimicrobial resistance continues to grow. A novel analysis framework of resistance risk is built in all One‐Health components, to estimate the risks of transmission routes of resistance genes between humans and other habitats, which can help to identify strategies for controlling the ongoing dissemination and antibiotic resistance.

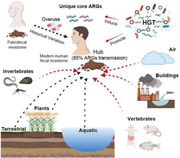

## 1 Introduction

Antimicrobial resistance jeopardizes the management of infectious diseases and has emerged as one of the leading public‐health crises of the 21st century. An estimated five million deaths were associated with bacterial antibiotic resistance in 2019,^[^ ^1^ ^]^ which is expected to increase to 10 million deaths per year by 2050, costing up to US$100 trillion globally.^[^ ^2^ ^]^

Commensal bacteria in the human gut comprise a complex and highly dense polymicrobial community known to be a reservoir of antibiotic‐resistance genes (ARGs), collectively known as the human resistome.^[^ ^3^ ^]^ The human resistome is dynamic, with its diversity and abundance affected by geography,^[^ ^4^ ^]^ age,^[^ ^5^ ^]^ body state,^[^ ^6^ ^]^ and living environment.^[^ ^7^ ^]^ How and when the global human‐gut resistome was assembled, and what factors led to interindividual variation, however, are unclear. We do know that resistance genes are ancient, originating long before the antibiotic era.^[^ ^8^ ^]^ ARGs of both clinical and agricultural importance have been found in remote environments with minimal anthropogenic impacts, such as the beta‐lactam and tetracycline resistance genes that were detected in 30, 000‐year‐old permafrost,^[^ ^8^ ^]^ isolated caves,^[^ ^9^ ^]^ Alaskan soils^[^ ^10^ ^]^ and glaciers.^[^ ^11^ ^]^ Recently, a survey of metagenomic data revealed that many ARGs can be detected in different habitats, such as beta‐lactam resistance genes, which were widespread across human and environmental habitats.^[^ ^12^ ^]^ The connections between humans, animals, plants and environments, highlighted in the One Health framework, clearly provide mechanisms for the ancient resistome to colonize the human microbiota.^[^ ^13^ ^]^

Various routes for the transmission of resistance genes to humans from other habitats have been demonstrated, such as aquatic‐human transmission from recreational swimming, surfing and aquatic products,^[^ ^13^ ^]^ and from soil via agricultural products.^[^ ^14^ ^]^ Understanding transmission routes between humans and other habitats is essential for controlling and inhibiting the dissemination of resistance genes. Assessing and comparing the risk of cross‐transmission between humans and different habitats, however, is difficult.

Horizontal gene transfer (HGT) of resistance genes is among the most important mechanisms of dissemination^[^ ^15^ ^]^ and could help us understand the characteristics and risks of the resistome under a One‐Health framework.^[^ ^16^ ^]^ Phylogenetic and ecological diversity are crucial barriers to limiting the horizontal transmission of antimicrobial resistance between humans and other habitats.^[^ ^17^ ^]^ ARGs could benefit if they can overcome these barriers to HGT, as do their potential recipients.^[^ ^18^, ^19^ ^]^ Understanding the HGT efficiency of ARGs when overcoming such barriers would be a key step toward assessing and controlling the risk of resistance.

The rapid development of high‐throughput sequencing technology and bioinformatics provides unprecedented opportunities to study the characteristics of global resistome.^[^ ^20^ ^]^ We used 3018 and 20 data sets of modern and ancient fecal metagenomes, respectively, to catalog the genes and potential driving factors that assembled the human core resistome. We then collected metagenomic data sets for seven external habitats to investigate the commonalities and differences between humans and external habitats’ resistome (Figure S1, Supporting Information). Finally, we catalogued the global shared resistome and used 10274 bacterial genomes isolated from human feces and external habitats to construct a global exchange network of ARGs. These data will inform risk assessments for resistance in a One Health framework and could identify strategies for controlling the ongoing dissemination and antibiotic resistance.

## 2 Results

### 2.1 Historical Variation in the Human Fecal Resistome

We collected a set of metagenomes from 20 samples of palaeofeces (1000‐2611 years old)^[^ ^21^, ^22^, ^23^ ^]^ and 3018 samples of modern‐human feces (ages from 0 to 90 years, uploaded to public databases from 2004 to 2018) from 23 countries across five continents (Table S1, Supporting Information). Principal coordinate analysis (PCoA) with Bray‐Curtis dissimilarity showed that resistomes from paleofeces were clearly separated from modern‐human feces (n = 768, healthy adults) (Adonis analysis, adjust *p* < 0.05; Figure S2A, Supporting Information). The paleofeces resistome was closest to Fiji (Figure S2B, Supporting Information), resulting from higher source contributions in paleofeces resistome to these countries by using fast expectation‐maximization for microbial source tracking (FEAST) (Figure S3A, Supporting Information). An average of 20.11% source contributions of paleofeces resistome to modern humans, of which 486 shared ARGs mainly conferred multidrug resistance (Figure S3B, Supporting Information).

**Figure 1:**
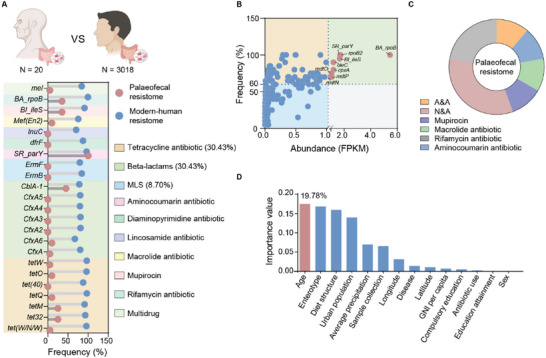
Historical variation and driving factor of human fecal core resistomes. A) We collected metagenomic data from palaeofeces (n = 20) and modern‐human feces (n = 3018), and found that the prevalence of modern‐human fecal core ARGs was extremely low in palaeofeces. B) and C) The dominant ARGs in palaeofeces (>1 abundance (reads per kilobase per million mapped reads, RPKM) and >60% frequency), and the associated classification. A&A, aminoglycoside and aminocoumarin antibiotic; N&A, nucleoside antibiotic and acridine dye. D) Machine learning with the random‐forest algorithm was used to determine the importance of optimal factors (VIFs < 5) driving the core resistomes. Age was the most important factor to drive the core resistomes in modern human feces. GNI, gross national income; VIFs: variance inflation factors.

Based on the core index (see “Extended methods” section), we identified a total of 22 core ARGs in modern human feces (Figure S4, Supporting Information), which mainly conferred resistance to tetracyclines and beta‐lactams. The prevalence of the core ARGs of modern humans was relatively low in paleofeces (**Figure** **1A**), whereas nucleoside antibiotic and acridine dye resistance genes dominated the paleofecal resistome (Figure 1B,C). This finding further highlighted the historical variation in human fecal resistome.

### 2.2 Factors in Driving the Variation of Human Core Resistome

The peak of the distribution of some core ARGs was distinctly lower than the medians of the country (Figure S4, Supporting Information), implying that the core resistome of modern humans presents obvious geographical differences (Figure S5, Supporting Information). We developed machine‐learning random forest regression models^[^ ^24^ ^]^ to identify the main factors that formed the core resistome in the human gut (n = 3018, factors see Table S2, Supporting Information). We selected the model with the highest accuracy rate (70%) for calculating the importance of 14 optimal factors (variance inflation factor < 5) in accounting for the core resistome (Figure S6, Supporting Information), and found that age was the most important factor with a 19.78% explanation rate (Figure 1D). PCoA showed that the pattern of core resistome gradually changed with age (Adonis analysis, *R* ^2^ = 0.086, adjust *p* < 0.05; Figure S7A, Supporting Information), in which infant and elder exhibited significant differences from adult (Adonis analysis, infant and adult: *R* ^2^ = 0.086, adjust *p* < 0.05; elder and adult: *R* ^2^ = 0.122, adjust *p* < 0.05, Figure S7B, Supporting Information).

### 2.3 Variation of the Core Resistome Across All Habitats

**Figure 2:**
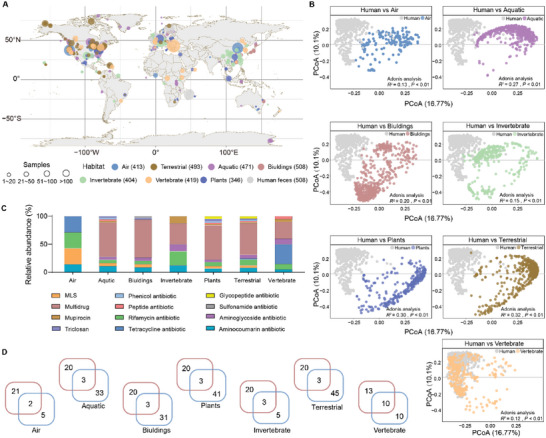
Variation of the core resistome across all habitats. A) We collected 3562 metagenomic datasets from the air (413), aquatic (471), buildings (508), invertebrate (404), plant (346), terrestrial (493), vertebrate, (419) and human feces (508). B) Principal coordinate analysis with Bray‐Curtis dissimilarity and Adonis analysis showed that the pattern of resistomes from different habitats is clearly separated from human feces. Red and blue circles indicate human feces and other habitats, respectively. C) The classification of the core resistome from different external habitats. D) Sharded and unique core ARGs between human feces and other habitat samples.

To gain insight into the role of habitats from a One Health perspective, we compiled a data set of 3562 metagenomic samples (Table S3, Supporting Information) and identified 2556 ARGs (Table S4, Supporting Information) from eight types of habitats (**Figure** **2A**). Buildings and plants harbored higher diversity (Shannon index) of resistome compared with other habitats (Figure S8A, Supporting Information). PCoA clearly separated the patterns of resistomes between human feces and other habitats (Adonis analysis, adjust *p* < 0.01; Figure 2B). Adonis analysis indicated that the structure of resistome in the air habitat was the most similar of all environmental habitats to human feces, and the variations of resistomes between habitats were consistent with their ecological relationships (Figure S8B, Supporting Information), for example, the plant resistome was similar to the terrestrial resistome.

We identified 7, 36, 34, 44, 8, 48, and 20 core ARGs from the air, aquatic, building, plant, invertebrate, terrestrial and vertebrate microbiomes (Figure S9,Supporting Information), respectively. The categories of ARGs vary in different habitats (Figure 2C). For instance, aquatic, building, plant and terrestrial habitats harbored core ARGs conferring multidrug resistance, and the core resistome of vertebrates was mainly classified into tetracycline, similar to that in human feces. The human fecal core resistome overlapped little with other habitats (Figure 2D), and their shared network showed a habitat specificity (Figure S10, Supporting Information), further implying that the core resistome of different habitats might reflect unique features.

### 2.4 Resistome Elements Shared Between Human Feces and Various Habitats

**Figure 3:**
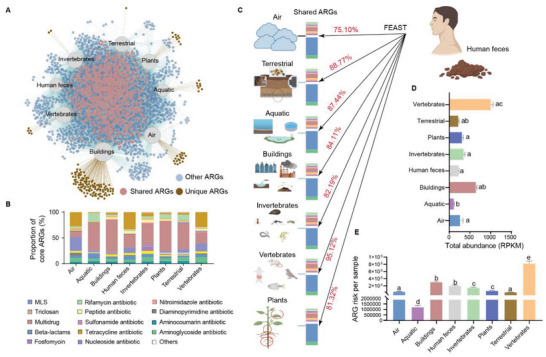
Shared pattern of resistomes across human feces and the other habitats. A) Network of resistomes shared between human feces and the external habitats (air, terrestrial habitats, aquatic habitats, buildings, invertebrates, vertebrates and plants). A total of 708 ARGs were shared among all habitats, and human feces had no unique ARGs. B) The abundance (RPKM) of shared ARGs varied considerably across habitats. C) Fast expectation‐maximization for microbial source tracking (FEAST) estimating the source contribution of human fecal resistome to the different habitats. These shared ARGs mainly conferred beta‐lactams resistance; most of the ARGs (≈85%) in other habitats were sourced from human fecal resistome. D) The total abundance of shared ARGs in human feces and the other habitats. E) The antibiotic resistance risk of samples from various habitats. Different letters represent significant differences between habitats (Kruskal‐Wallis test, adjust *p* < 0.05).

About 28% of the ARGs detected in the shared resistome network were shared across all habitats (n = 3562), and all ARGs in human feces were detected in other habitats (**Figure** **3A**). These shared ARGs mainly conferred multidrug and beta‐lactams resistance (Figure S11, Supporting Information). The category of shared elements differed between habitats (Figure 3B). FEAST estimations showed an average of 85% source contribution of human fecal resistome to every other habitat (Figure 3C; Figure S12, Supporting Information). The classification of shared ARGs was almost completely similar between human feces and other habitats, while the abundance of these elements between human and other habitats varied considerably (Figure 3D). Although vertebrates and buildings had the highest abundance of shared resistomes, air had the highest abundance of high‐risk ARGs, significantly higher than the other environmental habitats (aquatic and terrestrial habitats) (Kruskal‐Wallis test, adjust *p* < 0.05; Figure 3E).

### 2.5 Identification of ARG Hosts from Human Feces

**Figure 4:**
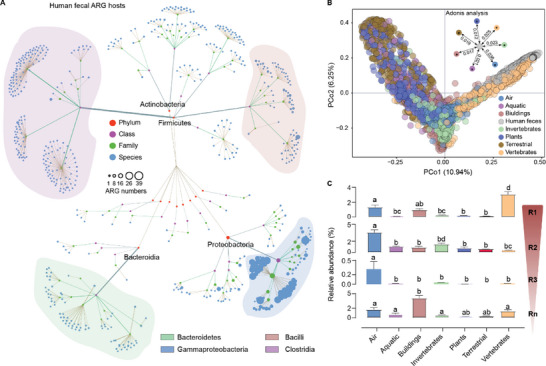
Habitat filtering for the ARG hosts. A) Phylogenetic taxonomic network of ARG hosts in human feces. A total of 720 ARG hosts (species level) were identified based on the 177134 metagenome‐assembled genomes (MAGs) of the human fecal microbiome collected from 33 countries across six continents compiled by Almeida et al^45^. The color and size of the circles indicate the taxonomic level and ARG numbers (per species), respectively, and the width of the lines indicates the richness of the taxonomic levels. *Clostridia*, *Gammaproteobacteria*, *Bacilli*, and *Bacteroidetes* were the main ARG hosts in human feces. B) Principal coordinate analysis with Bray‐Curtis dissimilarity showed the variation pattern of ARG hosts between human feces and other habitats. Adonis analysis indicates the ARG hosts from each habitat were significantly separated from human feces (“*”, adjust *p* < 0.05). C) The relative abundance of the pathogenic ARG hosts from human feces in the other habitats. Based on the database of Pathogen Host Interactions and the list of antibiotic‐resistant “priority pathogens” (World Health Organization), we defined the pathogenic ARG hosts as R1 (Priority 1: CRITICAL), R2 (Priority 2: HIGH), R2 (Priority 3: MEDIUM), and Rn (other human pathogens). Different letters represent significant differences between the relative abundance (Kruskal‐Wallis test, adjust *p* < 0.05).

Microbiomes determine the variation in resistomes and the risk of antimicrobial resistance.^[^ ^25^ ^]^ Based on our framework for identifying ARG hosts,^[^ ^26^ ^]^ we identified 332640 ARGs from the human‐gut metagenome‐assembled genomes (MAGs) associated with microbes (n = 177134) recovered by Almeida et al.^[^ ^27^ ^]^ A total of 38630 MAGs were identified as ARG hosts (including 720 species from 72 families) (Table S5, Supporting Information) (see **Figure** **4A** for their taxonomy). *Clostridia*, *Gammaproteobacteria*, *Bacilli* and *Bacteroidetes* were the main ARG hosts in human feces, especially *Enterobacteriaceae* and *Pseudomonadaceae* in the *Gammaproteobacteria*. These taxa are the most lethal antibiotic‐resistant bacteria (https://www.who.int/). Potentially high‐risk ARG hosts were also detected, such as *Hafniaceae*, *Moraxellaceae* and *Yersiniaceae*. We found 57 new ARG hosts by Blasting 145783 bacterial genomes collected from the database of the National Center for Biotechnology Information isolated from all habitats based on the above lists of ARG hosts (Table S6, Supporting Information). These data enriched the database of potentially antibiotic‐resistant bacteria.

### 2.6 Habitat Filtering for ARG Hosts

PCoA with Bray‐Curits dissimilarity showed that the patterns of human feces were significantly separated from those of the seven other habitats, respectively (Adonis analysis, adjust *p* < 0.05; Figure 4B). These metrics were generally higher in the microbiomes from air, buildings and vertebrates (Figure S13, Supporting Information). Based on the database of Pathogen Host Interactions and the list of antibiotic‐resistant “priority pathogens” from the WHO, we further defined the pathogenic ARG hosts as: Priority 1‐CRITICAL, Priority 2‐HIGH, Priority 3‐MEDIUM, and other human pathogens. The vertebrate and building habitats contained more‐abundant CRITICAL priority pathogens, and the air and invertebrate habitats contained the highest abundances of HIGH and MEDIUM priorities, respectively (Figure 4C). This finding indicated that the vertebrate, building, air, and invertebrate habitats pose serious threats to human health, because they harbor more‐abundance priority antibiotic‐resistant bacteria than do other habitats.

### 2.7 The pattern of Dissemination of Resistomes Across Human Feces and other Habitats

**Figure 5:**
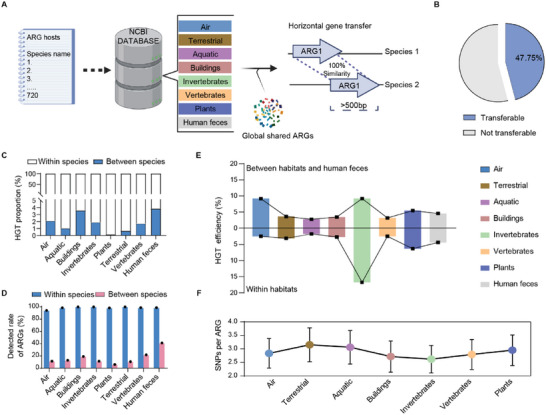
Pattern of dissemination of resistomes across human feces and the other habitats. A) Based on the list of 720 ARG hosts from human feces, we collected 10 274 bacterial genomes from the various habitats based on the database of the National Center for Biotechnology Information to construct the ARG exchange network with the global shared resistome (n = 400). We used Blast hits with 100% similarity and lengths >500 bp. B) We obtained 5555932 horizontal gene transfers (HGTs), including 2, 088998 genomic pairs, and 191/414 transferable ARGs. C) The proportions of HGT efficiency between and within species in the various habitats. D) The rate of detection of ARGs between and within species in the various habitats. E) We collected 2090 *E. coli* genomes to calculate the efficiency of transfer within species across the external habitats and human feces (See “Method”). The cross‐transmission routes between human feces and air, invertebrates and plants had higher HGT efficiencies within *E. coli* strains than within human feces; the efficiency of transfer within *E. coli* was higher in invertebrates and plants than the other external habitats. F) The number of single nucleotide polymorphisms (SNPs) for each transferable ARG in different routes of transmission. The average number of SNPs for transferable ARGs in genomic pairs from cross‐transmission routes between human feces and air, invertebrates and plants was lower than for the other habitats.

To examine the transmission of ARGs between the environment and humans, we collected 10274 genomes of specific bacteria isolated from human feces and seven habitats based on the list of ARG hosts identified from the database of the National Center for Biotechnology Information (Table S7, Supporting Information). These genome data had obvious geographical differences, but using them was still an effective and convenient approach to studying the evolution of global species.^[^ ^28^ ^]^ We then constructed an ARG exchange network, with a total of 5, 555, 932 HGTs with 2, 084, 607 genomic pairs (**Figure** **5A**). Almost half of the used ARGs (n = 400) were transferable (Figure 5B), mainly ARGs that conferred resistance to multidrug and beta‐lactam antibiotics (Figure S14A, Supporting Information). These ARGs were associated with more types of mobile genetic elements (within 5 kb upstream and downstream of the ARG) than non‐transferable ARGs across the various habitats (Figure S14B, Supporting Information). We calculated the efficiency of transfer of each transferable ARG within and between species in various habitats and found that HGT efficiency was significantly higher within than between species (Figure 5C,D).

Most ARGs could be transferred between species across habitats and some unique ARGs are only transferred horizontally between species within human feces (Figure S15, Supporting Information). HGT efficiency between species was higher for the terrestrial and vertebrate habitats than for human feces. Because the terrestrial habitats harbor the most diverse and complex microbiome on Earth,^[^ ^28^ ^]^ intensive species interaction increases HGT of ARGs between soil microbes. The high consumption of antibiotics in animal husbandry provides continuous selection for the vertebrate microbiome to promote ARG enrichment and dissemination.

### 2.8 HGT Efficiency and Evolution of Shared ARGs in Various Cross‐transmission Routes

We selected the most common species of antibiotic‐resistant bacteria, *E. coli*, which had the highest frequency of isolation in all habitats (n = 2090, HGT count = 685414; Table S8, Supporting Information). The HGT efficiency within *E. coli* strains was higher in air (9.15%), invertebrates (16.94%), and plants (6.48%) than in human feces (4.54±0.3%) (Figure 5E). The average number of single nucleotide polymorphisms (SNPs) of transferable ARGs in genomic pairs was lower in the cross‐transmission routes between human feces and air, invertebrates and plants, supporting the above result (Figure 5F, Table S9, Supporting Information). The number of SNPs transferred per ARG was positively associated with HGT efficiency, especially in the cross‐transmission routes between human feces and air and invertebrates (Ordinary least squares linear regression analysis, air‐: *R* ^2^ = 0.747, adjust *p* < 0.0001; plant‐: *R* ^2^ = 0.115, adjust *p* < 0.05; Figure S16, Supporting Information), implying that transferable ARGs participating in an earlier HGT had a stronger potential for transmission across human and other habitats.

The efficiency of transfer of transferable ARGs within *E. coli* strains from cross‐transmission routes was diverse (Figure S17A, Supporting Information), e.g. the efficiency of transfer of peptide‐resistance genes was higher in the cross‐transmission routes between human feces and invertebrates and plants, and the efficiencies of transfer of the aminocoumarin‐resistance genes were higher in the cross‐transmission routes between human feces and aquatic habitats, terrestrial habitats and buildings‐ (Figure S17B, Supporting Information). Multidrug‐resistance genes had the highest HGT efficiency across all cross‐transmission routes.

## 3 Discussion

ARGs are found in all microbial genomes,^[^ ^8^ ^]^ representing a cluster of functions that have been co‐opted to produce resistant phenotypes.^[^ ^29^ ^]^ Understanding these resistance genes and their transmission is a critical link in the One Health framework for addressing the antibiotic crisis. The presence of ARGs in the microbiome often may not increase their survivability in the environment without the selection pressure provided by antibiotics.^[^ ^28^ ^]^ Such genes are unlikely to perform their original functions when they are in a new genomic background.^[^ ^13^ ^]^

Only ARGs spread to the human microbiome cause serious health risks. Humans are thus a key factor in the One Health framework for understanding the global resistome. ARGs and associated bacterial hosts often cross habitat boundaries,^[^ ^11^ ^]^ so the resistomes from these habitats (environmental, buildings, animal and plant), have been considered as a primary source or sink of the clinical resistome.^[^ ^30^ ^]^

We annotated 6092 metagenomic samples from palaeofeces, modern‐human feces, and seven other habitats to identify ARGs and gain a view of the historical variation and dissemination of the human fecal resistome across habitats. The resistome structure of external habitats differed significantly from the human fecal resistome, likely driven by biotic and abiotic factors in the various habitats.^[^ ^31^ ^]^ We subsequently identified a unique core resistome of human feces, which was extremely different from those in the other habitats, likely due to combinations of microbial population structure, the horizontal acquisition of resistance genes and ongoing selection by antimicrobial agents. For example, we found that the main bacterial hosts of the ARGs varied dramatically across habitats (Figure S18, Supporting Information).

Most of the ARGs (≈85%) from each habitat, however, were transmitted with the human fecal resistome, indicating that human feces are now a hub of the global resistome. This development was not an accident, because modern human activity disseminates microbes on a global scale,^[^ ^32^ ^]^ intensifying the potential for transmitting ARGs between humans and other habitats. Meanwhile, the efficiency of the horizontal transfer of ARGs between *E. coli* strains was significantly higher in cross‐transmission routes between human feces and air, invertebrates and plants than within human feces, potentially associated with the recency of HGTs within *E. coli* strains. Some ARG hosts that frequently emerge in human and other habitats can also frequently transmit across habitats after phylogenetic barriers are overcome, further highlighting human feces as a hub of ARG accumulation. We used molecular clocks to determine that ARGs with earlier HGTs had higher efficiencies of transmission across habitats, highlighting their importance for linking the modern global antibiotic resistome. These genes should be added to the list of ARGs with the highest health risk.

Most importantly, the human fecal resistome has been formed by recent ARG transmissions across habitats, indicated by the significant differences between the palaeofecal and modern samples, with the palaeofeces harbored more multidrug ARGs. Although the palaeofecal samples were limited in number and do not fully represent the global ancient human resistome, nevertheless they still generated useful insights, despite the sampling and technical limitations. The machine‐learning analysis indicated that the core ARGs were mainly driven by age, diet and enterotype. Paying more attention to the antibiotic resistance in early life might be important,^[^ ^33^ ^]^ and dietary modifications may be an effective strategy for managing the burden of antimicrobial resistance.

Managing antimicrobial resistance has also been focused on the risk of transmission across the external environment and human feces.^[^ ^30^ ^]^ Our results showed that the built habitat, especially sewage treatment plants and subways, contained a higher abundance of ARGs and pathogens, presenting a higher health risk to humans. Consequently, vertebrate and built habitats constitute a significant reservoir of ARGs and antibiotic resistance bacteria. We thus urge health protection in built environments, and hygienic contact with farmed animals or pets.^[^ ^34^ ^]^ Interestingly, the HGT efficiency of ARGs between humans and invertebrates was 373% of that within the human habitat, but the risk of antimicrobial resistance in invertebrates continues to be rarely studied. Many invertebrates are favored foods by humans worldwide, especially raw food that could be able to direct contact with humans, posing a serious risk of resistance. Most importantly, we found that the air habitat not only harbored the most abundant high‐risk ARGs and contained more abundant priority antibiotic‐resistant pathogens,^[^ ^35^ ^]^ but also had a high HGT efficiency of ARGs cross‐transmission. This may be due to air being an ecosystem capable of long‐distance transport and with direct exposure to humans in any region.^[^ ^35^ ^]^ We thus stress that these cross‐transmission routes are of greater concern in the future, and some control management should be highlighted, such as enhancing people's awareness of protection in polluted areas or cities and strengthening the safety of raw food.

## 4 Conclusion

**Figure 6:**
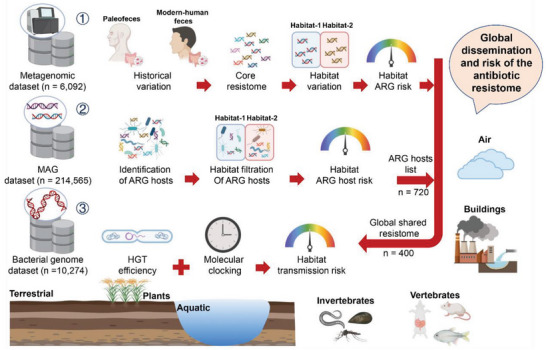
Overview of this study. We collected three datasets including metagenomics (n = 8061), metagenome‐assembled genomes (MAGs, n = 177134) and bacterial genomes (n = 10274) from paleofeces, modern‐human feces, buildings, air, aquatic, and terrestrial, plants, vertebrates and invertebrates. First, we determined the historical and habitat variations of human fecal resistome, identified core resistome from various habitats and quantified the ARGs risk of different habitats by using a metagenomic dataset. Second, we identified the ARG host (n = 720) in human feces by using MAG datasets and evaluated their risk in various habitats. Finally, we calculated the HGT efficiency of ARGs (n = 400) across various habitats to determine the transmission risk by using the bacterial genomes. The ongoing process of acquiring genes by HGT has overcome and altered the ecological and phylogenetic barriers to such horizontal transfers, probably by the co‐selection of promiscuous mobile genetic elements that carry resistance genes as cargo.

Systematically studying the resistome in specific regions under a One Health framework is necessary. Metagenomic sample collection, storage, transportation, DNA extraction, sequencing methods, and sequencing depth from different independent studies all lead to analysis biases in investigating global resistome. In this study, we carried out detailed sample information collection, built strict filtering sample standards, and controlled the data quality strictly, to alleviate the impact of the data deviation. Despite the data being heterogeneous, our study is nevertheless the most comprehensive study to date focusing on the human fecal resistome under a One Health approach (**Figure** **6**), and provides clear analytical and research perspectives for future studies. We developed a new study framework of the antibiotic resistome under a One‐Health perspective and demonstrated that widespread use of antimicrobial agents might have co‐opted antibiotic resistance genes from various environments into the modern human microbiome, where they have been fixed and have increased in abundance. Monitoring the formation of the human resistome in early life, and controlling air and animal cross‐transmission routes of antibiotic resistance can contribute to the context of WHO‐identified targets of antibiotic resistance prevention.

## 5 Experimental Section

### Data Collection

Data sets of metagenomes, metagenome‐assembled genomes (MAGs) and completed genomes was used to evaluate the variation between the character and structure of resistomes in humans and other habitats and to depict their system of dissemination by HGT. Metagenomic datasets available across the NCBI, EBI and other databases, and downloaded 6092 metagenomic samples using IBM Aspera Connect (v4.1.1) for determining the structure and abundance of ARGs across paleofeces, modern‐human feces (from 23 countries on five continents) and other habitats, including buildings, environment (air, terrestrial and aquatic habitats), animals (invertebrates and vertebrates) and plants were comprehensively searched. To maintain quantitative balance and representativeness of metagenomic data from different habitats, the data were strictly filtered according to the following standards: 1) Ensuring the public availability of metagenomic samples. 2) Making the sample size of each habitat the same order of magnitude; 3) Keeping the number of independent studies equal; 4) Maintaining a uniform distribution of samples at spatial scales; 5) Obtaining more types of sub‐habitats from each habitat. 6) Selecting fecal samples of healthy adults that were associated with metadata.

MAGs of 37431 and 177134 were downloaded from the IMG/M portal with the permission of Nayfach et al^[^ ^27^ ^]^ and Almeida et al,^[^ ^26^ ^]^ respectively. To delineate the HGT network and the evolutionary age of the global shared resistome (n = 400), the genomes of specific bacteria was downloaded isolated from human feces (n = 5912), air (n = 99), terrestrial habitats (n = 861), aquatic habitats (n = 631), buildings (n = 1178), invertebrates (n = 140), vertebrates (n = 813) and plants (n = 640) from the NCBI database based on the species lists of ARG hosts (identified from human feces). An index of the human‐health risk and the rank of each ARG was calculated from the previous study.^[^ ^36^ ^]^ Detailed information for these data sets were provided in Figure S1 and Tables S1 and S2 (Supporting Information).

### Taxonomic Annotation and Calculation of ARG Abundance

FastQC (v0.11.5; https://github.com/s‐andrews/FastQC) was used to check the quality of the raw metagenomic data that were further trimmed and filtered for quality using Trimmomatic (v0.36).^[^ ^37^ ^]^ ARGs were annotated in the Comprehensive Antibiotic Research Database (CARD 2020)^[^ ^38^ ^]^ using reads by RGI (v5.1.1) with default parameters at the metagenomic level. Reads were mapped to the ARGs in each sample using BWA (v0.7.13), and unmapped reads were removed using Samtools (v1.3.1).^[^ ^39^ ^]^ The abundances of the ARGs were calculated as reads per kilobase per million mapped reads, based on the number of mapped reads and the lengths of genes using a script available at GitHub (see “Code availability”). The ARGs were manually reclassified based on the drugs to which they conferred resistance, as detailed in the previous study.^[^ ^12^ ^]^

### Calculation of ARG HGT Efficiencies

To determine the HGT potential of each ARG in more detail, the absolute number of distinct Blast hits were considered between two genomes and the total number of possible pairings of each ARG in genomic pairs for calculating the efficiency of transfer of each transferable ARG within and between species. The number of possible pairings within species in the same habitat (S_Within_) was calculated as:

(1) $$S_{\mathrm{Within}}=\sum_{i=1}^{n}\left(n_{i}-1\right)+\frac{\left(n_{i}-1\right)\times \left(n_{i}-2\right)}{2}$$

where ni was the number of genomes of the same species in the same habitat.

The number of all possible pairings in the same habitat (S_All_) was calculated as:

(2) $$S_{\mathrm{All}}=\left(N-1\right)+\frac{\left(N-1\right)\times \left(N-2\right)}{2}$$

where N was the number of genomes of the same species.

The efficiency of transfer of transferable ARGs between species in the same habitat (ES_Between_) was calculated as:

(3) $${\mathrm{ES}}_{Between}=\frac{H_{TR}}{\left(S_{All}-S_{Within}\right)}\times A$$

where HTR and A were the number of HGTs between species and the number of transferable ARGs in the same habitat, respectively.

The efficiency of transfer of transferable ARGs within species in the same habitat (ES_Within_) was calculated as:

(4) $${\mathrm{ES}}_{Within}=\frac{H_{TR}}{S_{Within}}\times A$$

where HTR and A were the numbers of HGTs between species and the number of transferable ARGs in the same habitat, respectively.

The efficiencies of transfer of transferable ARGs within (ET_Within_) and between (ET_Between_) species across routes of transmission were calculated as:

(5) $${\mathrm{ET}}_{Within}=\frac{H_{Within}}{h\times f}\times B$$

(6) $${\mathrm{ET}}_{Between}=\frac{H_{Between}}{h\times f}\times B$$

where H_Within_ and H_Between_ were the numbers of HGTs within and between species across routes of transmission, respectively, h and f were the numbers of genomes in habitats and human feces, respectively, and B was the number of transferable ARGs across routes of transmission.

### Identification of Core Resistomes from Microbiomes Based on The Analysis of Metadata

Unlike the traditional identification of core members in microbial communities, a method that simultaneously considered the abundance, prevalence and rate of detection (from all independent studies) of members (including functional groups and microbiomes) in a specific habitat for quantifying their core potentials based on the previous study was developed.^[^ ^40^ ^]^ This process considered the detection rate and abundance of ARGs in specific habitats or communities. The detection rate was considered for all independent experiments in the metadata, making the identification of a core resistome for the specific habitats more universal.

Indicators for membership of the core resistome for each ARG in each habitat were based on the frequency and relative abundance from all metagenomic samples and the rate of detection from all independent studies (≥50% ARGs detected in each study). All indicator values were normalized to avoid the effects of weighting the original numerical values for calculating the core index (CI) of each ARG in the various habitats:

(7) $$CI_{i}=RA_{i}\times \frac{F_{i}\times f_{i(>50\%)}}{N_{i}\times n_{i}}$$

where RA_i_ and F_i_ were the relative abundance (averages) and the number of ARGs from all samples in habitat *i*, respectively, N_i_ was the number of samples in habitat *i*, f_i_ was the number of ARGs with frequencies >50% in habitat *i* in each independent study and n_i_ was the number of independent studies of habitat *i*. Considering the heterogeneity and sequencing depth limitations of metadata analysis, the RA > 1%, F/N > 60% and f/n > 60% (CI > 0.1) were set as the screening threshold for core resistome. Moreover, when using this formula with CI > 0.1, F/N > 60% should be ensured at the same time to avoid uneven sample numbers of different independent experiments in metadata.

### Identification and Pathogenic Classification of ARG Hosts Based On The Analysis of MAGs

MAGs of 177134 associated with the human‐gut microbiota in CARD 2020,^[^ ^38^ ^]^ 89256 of which were used for detecting ARGs were annotated. The MAGs and ARG contigs were taxonomically assigned using Kraken2 (v2.1.2)^[^ ^36^ ^]^ with the default parameters based on the National Center for Biotechnology Information Reference Sequence Database; ARG contigs >10 kb and ensured that their taxonomic affiliation coincided with that of the ARG‐containing MAGs. were considered.^[^ ^12^ ^]^ This method removes some false‐negative ARG‐host information but was still one of the best methods for identifying ARG hosts based on metagenomic data and can accurately identify a large amount of information about unisolated ARG hosts. A total of 38630 MAGs were identified as ARG hosts, which were taxonomically classified into 720 species from 72 families.

According to the taxonomic information of the identified ARG hosts and the Pathogen Host Interactions database^[^ ^41^ ^]^ and the list of antibiotic‐resistant “priority pathogens” recognized by the World Health Organization (https://www.who.int/news/item/27‐02‐2017‐who‐publishes‐list‐of‐bacteria‐for‐which‐new‐antibiotics‐were‐urgently‐needed), these ARG hosts were classified into Priority 1‐CRITICAL, Priority 2‐HIGH, Priority 3‐MEDIUM and other human pathogens.

### Number and Ages of HGTs

Blast hits with 100% similarity and lengths >500 bp was used to identify recent HGTs in the global shared resistome (ARGs >500 bp, n = 400) between pairs of species.^[^ ^42^ ^]^ To understand the efficiency of the transfer of each ARG more systematically between pairs of genomes, the number of potential pairings between each genome and gene and the number of HGTs as the number of between‐species genomic pairs that shared at least one HGT of each ARG (500 bp + HGT) was also considered. the number of SNPs were also calculated for HGT per ARG between genomic pairs. Assuming a genome size of ≈10^6^ bp and a molecular clock of one SNP/genome/year, HGTs >500 bp with >99% similarity was consistent with transfers that occurred between 0 and 10000 years ago.^[^ ^42^ ^]^

### Statistical Analysis and Visualization

Data were presented as means ± Standard Error of Means. For differences using multiple methods were tested, as described in the main text. Kolmogorov‐Smirnov test was performed in Graphpad Prism 8 and calculated that the different nested dataset (ARGs and microbial abundance) in various groups do not conform to the normal distribution. Mann‐Whitney test and Kruskal‐Wallis test were used to pairwise comparisons in two or more groups by performing in Graphpad Prism 8. Linear mixing model and least squares linear regression were built in SPSS Statistics (v20.0.0) and generated by using Graphpad Prism 8. Principal co‐ordinates analyses and Adonis tests were performed and calculated using R (v3.6.3), and heatmaps were produced using TBtools (v1.113) and R (v3.6.3). The network analysis using Gephi v0.9.2 and Cytoscape v3.8.2. Venn diagrams were generated using EVenn (http://www.ehbio.com/test/venn; v1.0) were performed. Partitioning around medoids was used to determine the enterotypes based on Jensen–Shannon divergence^[^ ^43^ ^]^ (Table S3, Supporting Information). Data figures were created using Graphpad Prism 8 and R (v3.6.3), and Adobe Illustrator 2020 was used for formatting the figures. All schematic diagrams and art elements were drawn using BioRender (https://app.biorender.com/; v1.0) with full publishing rights. Fast expectation–maximization microbial source tracking (FEAST) was used for estimating the source proportion of human fecal resistome to each habitat and was conducted with the R package “FEAST” and visualized using Graphpad Prism 8. See supplemental information for details.

## Conflict of Interest

The authors declare no conflict of interest.

## Supporting information

Supporting Information

Supplemetary Table S1

Supplemetary Table S2

## Data Availability

The data that support the findings of this study are available from the corresponding author upon reasonable request.;
