# Supplementary material for: Metagenomic Insight into The Global Dissemination of The Antibiotic Resistome
Source: Adv Sci (Weinh). 2023 Oct 23;10(33):2303925. doi: 10.1002/advs.202303925 (PMC10667823; doi:10.1002/advs.202303925)
Supplement: Supplementary file 1 — Supporting Information [file ADVS-10-2303925-s002.pdf]

ADVANCED SCIENCE
Open Access

Supporting Information

for *Adv. Sci.*, DOI 10.1002/advs.202303925

Metagenomic Insight into The Global Dissemination of The Antibiotic Resistome

*Qi Zhang, Nuohan Xu, Chaotang Lei, Bingfeng Chen, Tingzhang Wang, Yunting Ma, Tao Lu, Josep Penuelas, Michael Gillings, Yong-Guan Zhu, Zhengwei Fu and Haifeng Qian**

Supplemental Information

Metagenomic insight into the global dissemination of the antibiotic resistome

Qi Zhang, Nuohan Xu, Chaotang Lei, Bingfeng Chen, Tingzhang Wang, Yunting Ma, Tao Lu, Josep Penuelas, Michael Gillings, Yong-Guan Zhu, Zhengwei Fu, Haifeng Qian[*]

Q. Zhang, N. Xu, C. Lei, B. Chen, T. Lu, H. Qian, Z. Fu

College of Environment, Zhejiang University of Technology, Hangzhou 310032, P. R. of China

E-mial: hfqian@zjut.edu.cn

T. Wang, Y. Ma

Key Laboratory of Microbial Technology and Bioinformatics of Zhejiang Province, Hangzhou 310012, P. R. of China

J. Penuelas

CSIC, Global Ecology Unit CREAF-CSIC-UAB, Bellaterra, Barcelona 08193, Catalonia, Spain

J. Penuelas

CREAF, Campus Universitat Autònoma de Barcelona, Cerdanyola del Vallès, Barcelona 08193, Catalonia, Spain

M. Gillings

ARC Centre of Excellence in Synthetic Biology, School of Natural Sciences, Macquarie University, NSW 2109, Australia

Y. Zhu

Key Laboratory of Urban Environment and Health, Institute of Urban Environment, Chinese Academy of Sciences, Xiamen 361021, P. R. of China

Y. Zhu

State Key Laboratory of Urban and Regional Ecology, Research Center for Eco-environmental Sciences, Chinese Academy of Sciences, Beijing 100085, P. R. of China

Z. Fu

College of Biotechnology and Bioengineering, Zhejiang University of Technology, Hangzhou 310032, P. R. of China.

Supplemental Figures

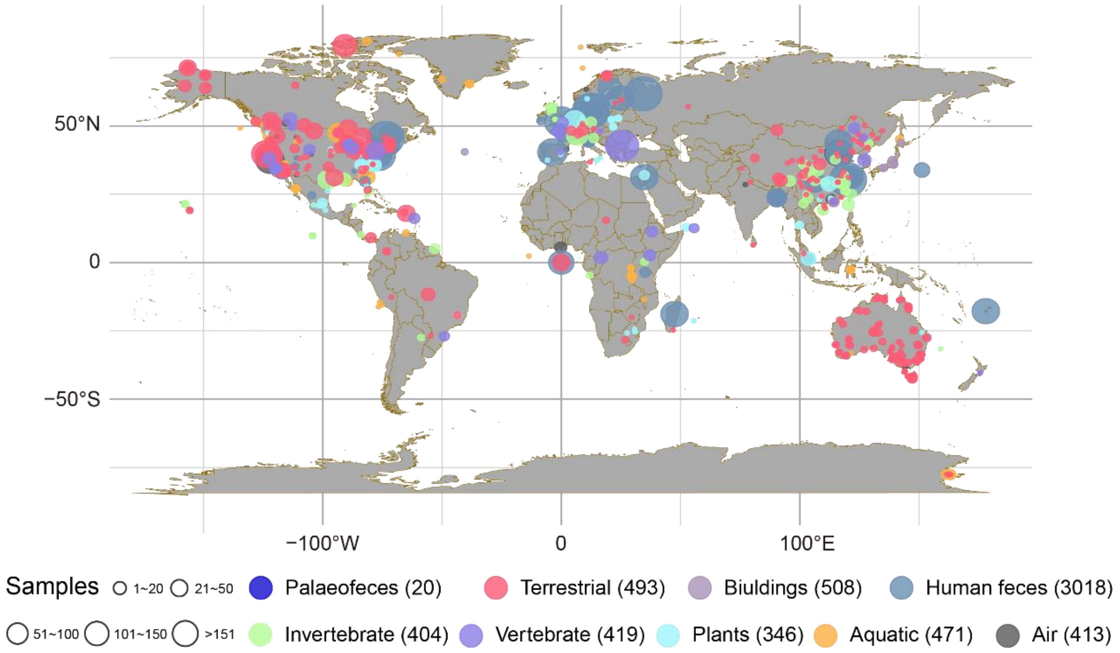

Figure S1. Geographic distribution of metagenomic sequencing data. Samples with unclassified locations are indicated as 0° latitude and 0° longitude. Circle size indicates sample size. More details are presented in Tables S1 and S2.

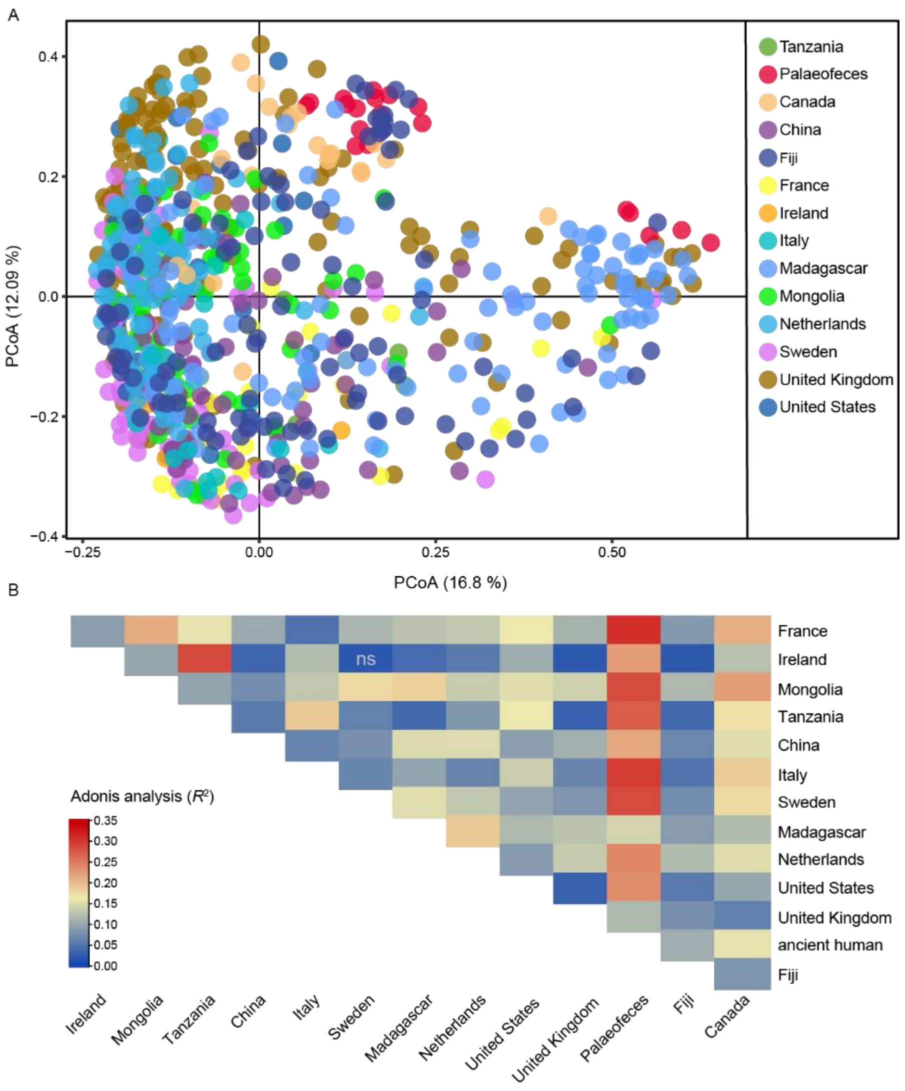

Figure S2. Variation of resistome in paleofecal, modern-human fecal samples. (**A**) Principal coordinate analysis with Bray-Curtis dissimilarity shows that the pattern of resistomes from paleofeces is clearly separated from modern human feces. (**B**) Heatmap shows the dissimilarity of resistome among paleofeces and modern-human feces from various countries using Adonis analysis. Color gradation indicates the R^2 values in Adonis analysis. "ns" represents no significant differences between countries (Adonis analysis, adjust $p < 0.05$).

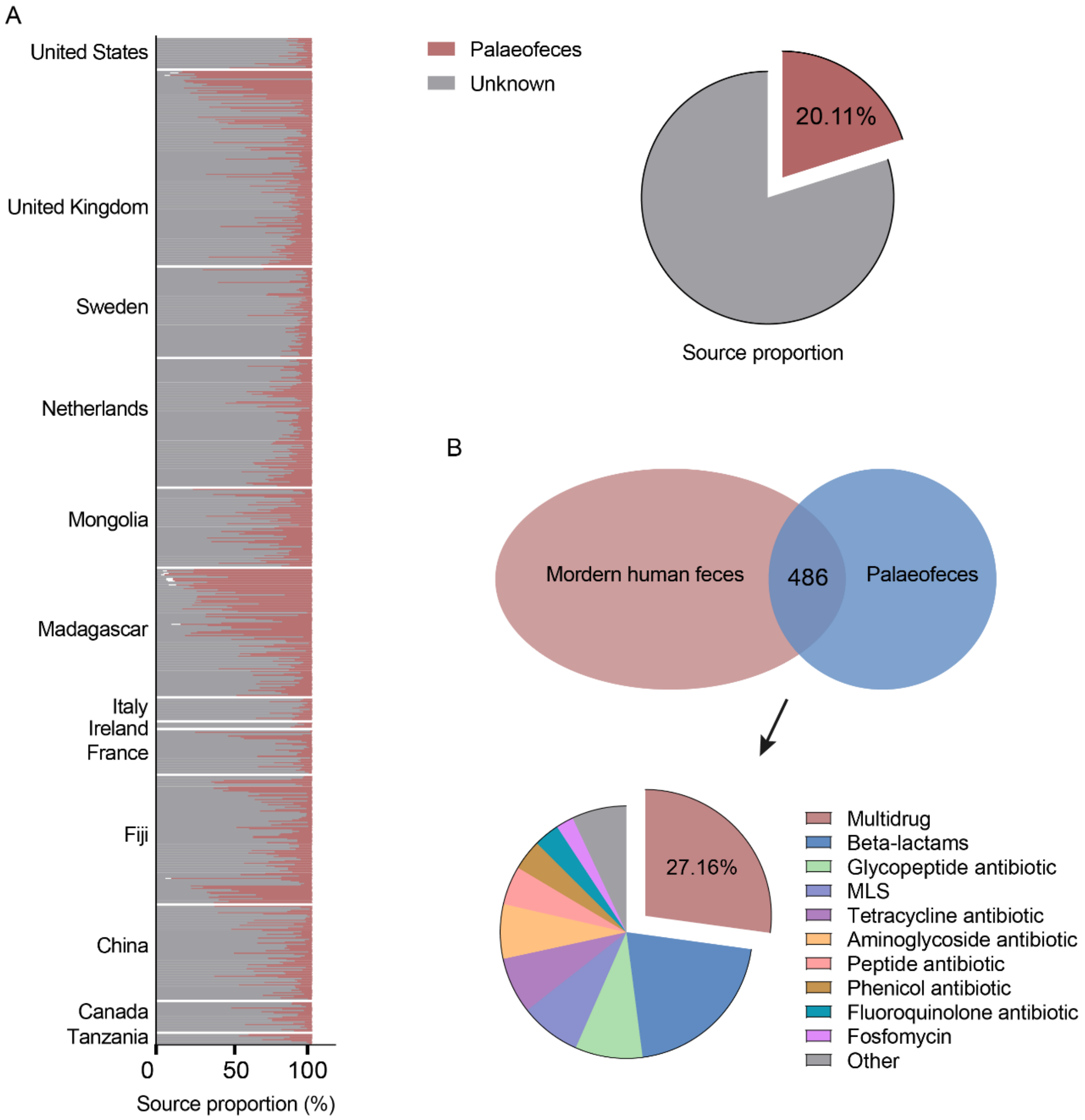

Figure S3. FEAST estimations of palaeofecal resistome contribution to the human feces resistome. (**A**) Source proportion of palaeofecal resistome in different countries modern human fecal resistome. A total of 20.11% modern human fecal resistome was sourced from palaeofecal resistome. (**B**) The shard ARGs and associated classification between palaeofecal and modern human fecal resistome.

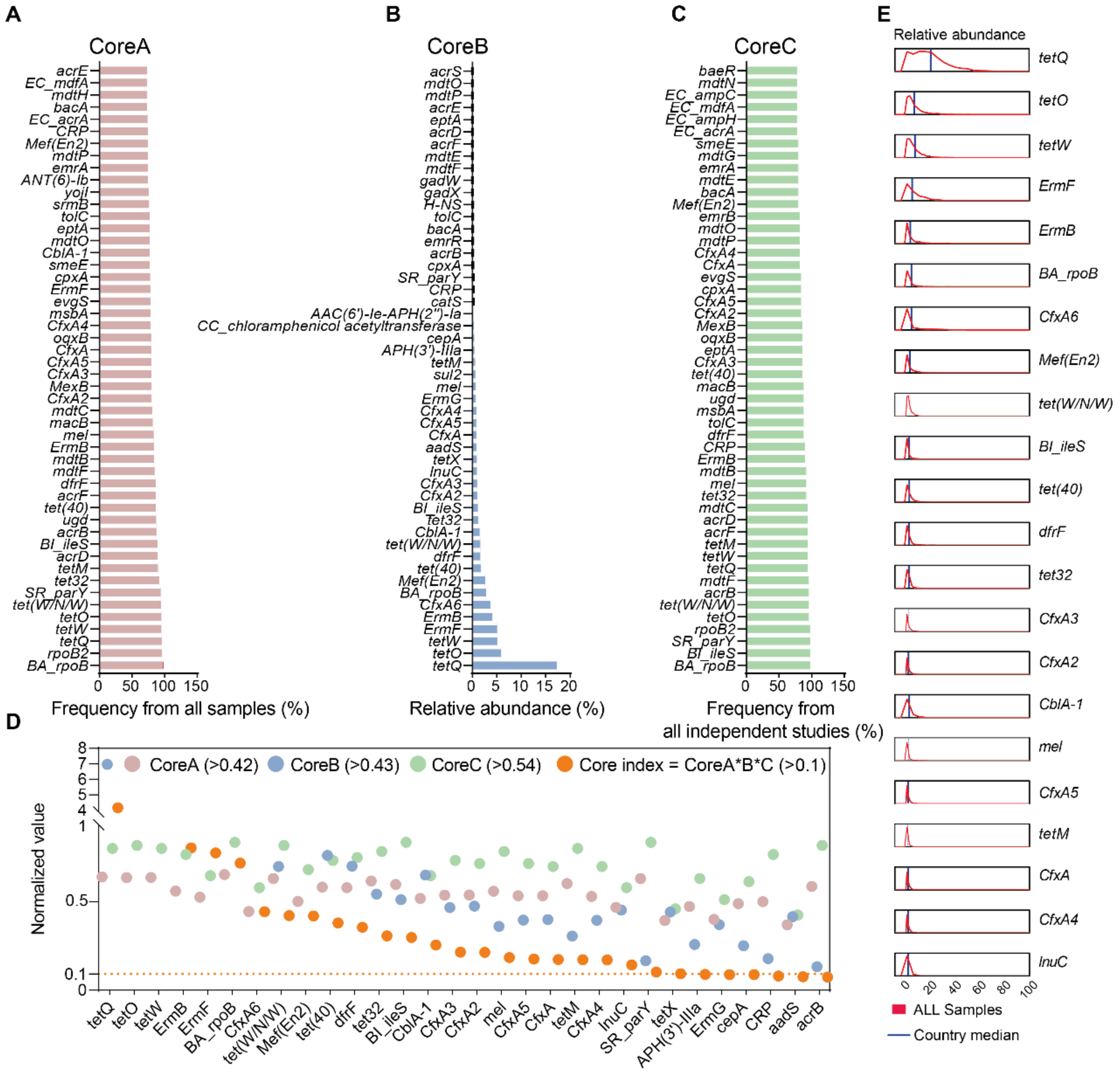

Figure S4. Identification of the core resistome in human feces. (**A**) and (**B**), Frequency (Core A) and relative abundance (Core B) of each ARG from all human fecal metagenomic samples. (**C**) Frequency of ARGs from all independent studies (Core C, N = 49). Rate of detection of ARGs (≥50%) in each study. (**D**) The calculation of the core index (CI = A×B×C) for each ARG. A threshold of CI > 0.1 and CoreC > 0.54 (60%) was used for identifying the core resistome. All values on the graph are normalized (rang 0 to 1). (**E**) The prevalence and distribution of relative abundances of the core ARGs from all samples. The peak of the distribution of some core ARGs was distinctly lower than the country medians.

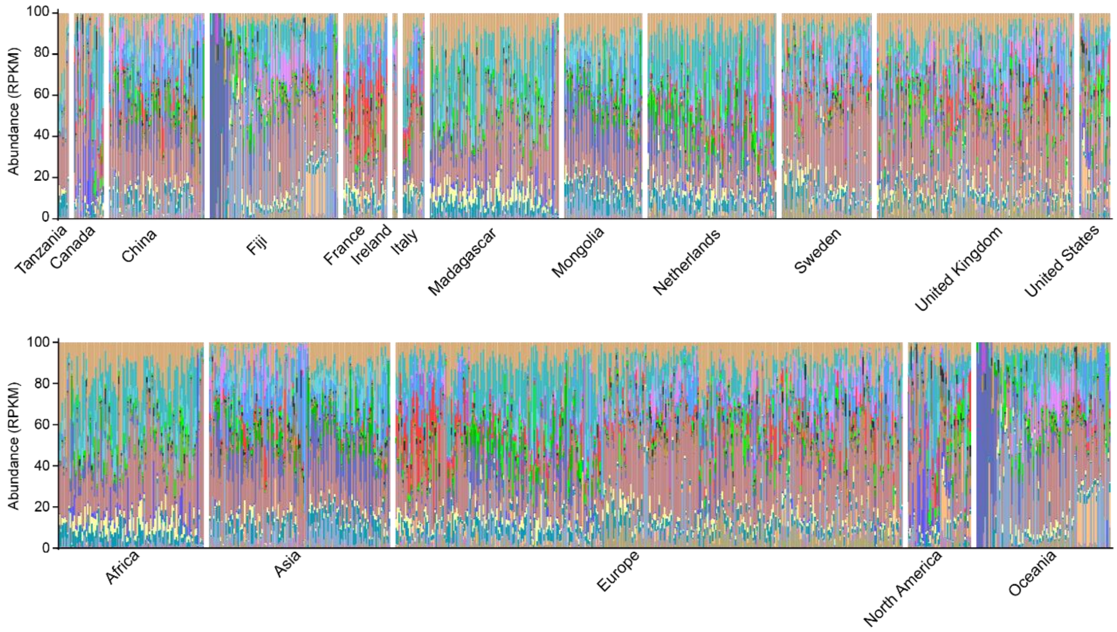

Figure S5. Fingerprint profile of global human fecal core resistome. The composition of core resistome in various countries and continents. RPKM: reads per kilobase per million mapped reads.

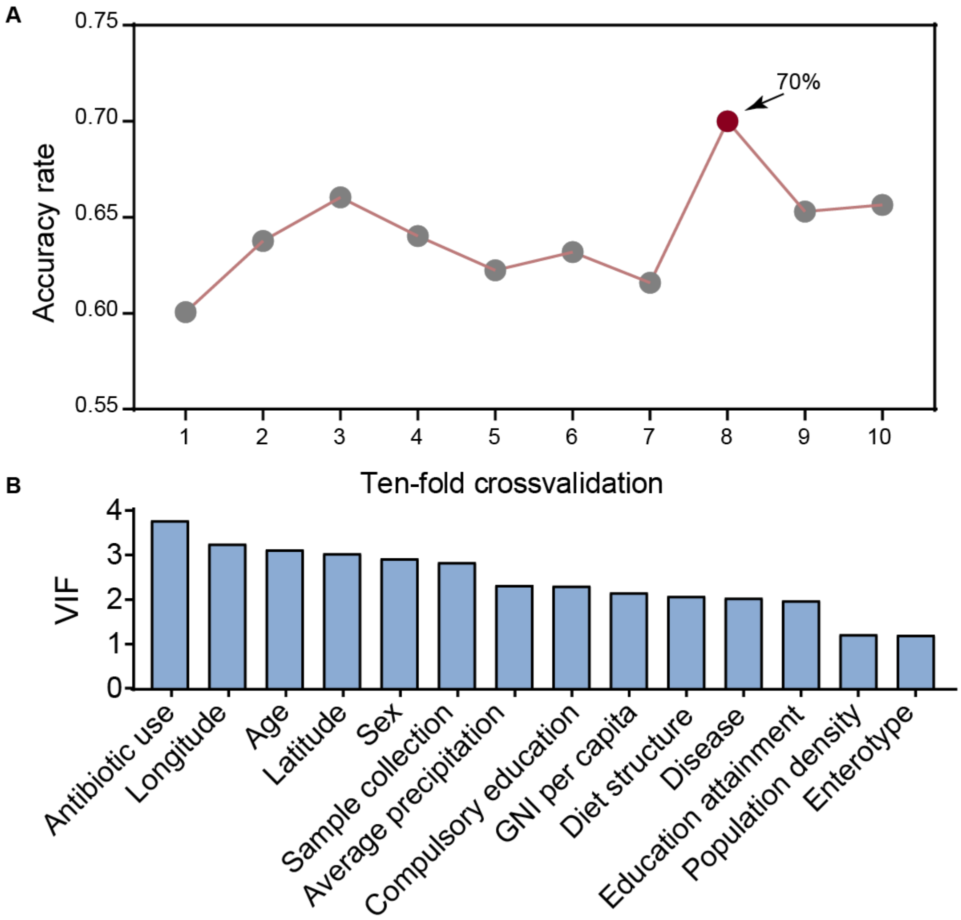

Figure S6. Investigation of the main factors affecting the core resistomes in modern human feces. (A) The high accuracy rate (>70%) of machine learning random forest (regression model) verified the correlation of these factors for the core resistomes based on 10-fold cross-validation. **(B)** The VIF of factors constructed machine-learning model. VIF: variance inflation factor.

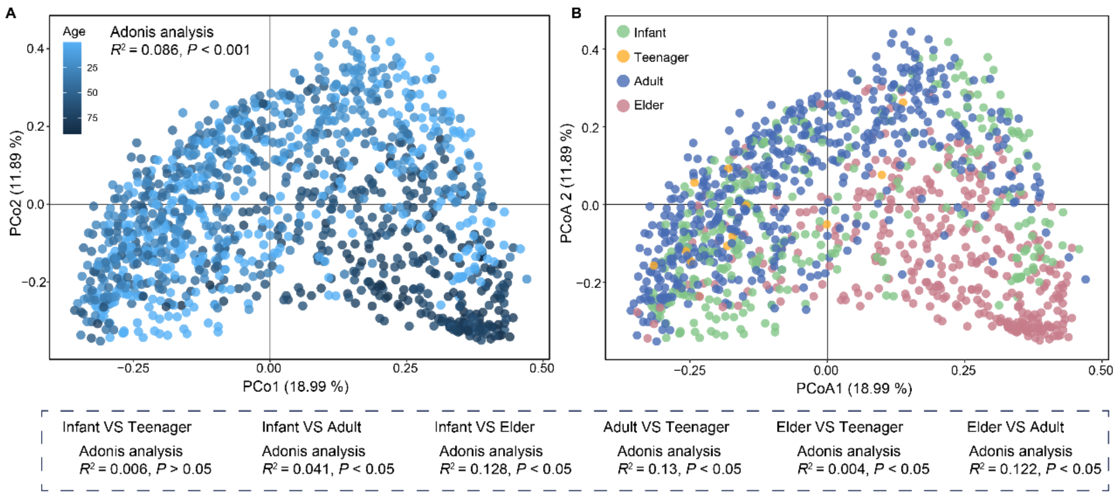

Figure S7. The structure of human resistome at different ages. (**A**) and (**B**) PCoA shows the structure of human resistome was significantly separated by age. Color gradation indicates the age of individuals. Green, yellow, blue and red indicate the infant, teenager, adult and elder fecal resistome.

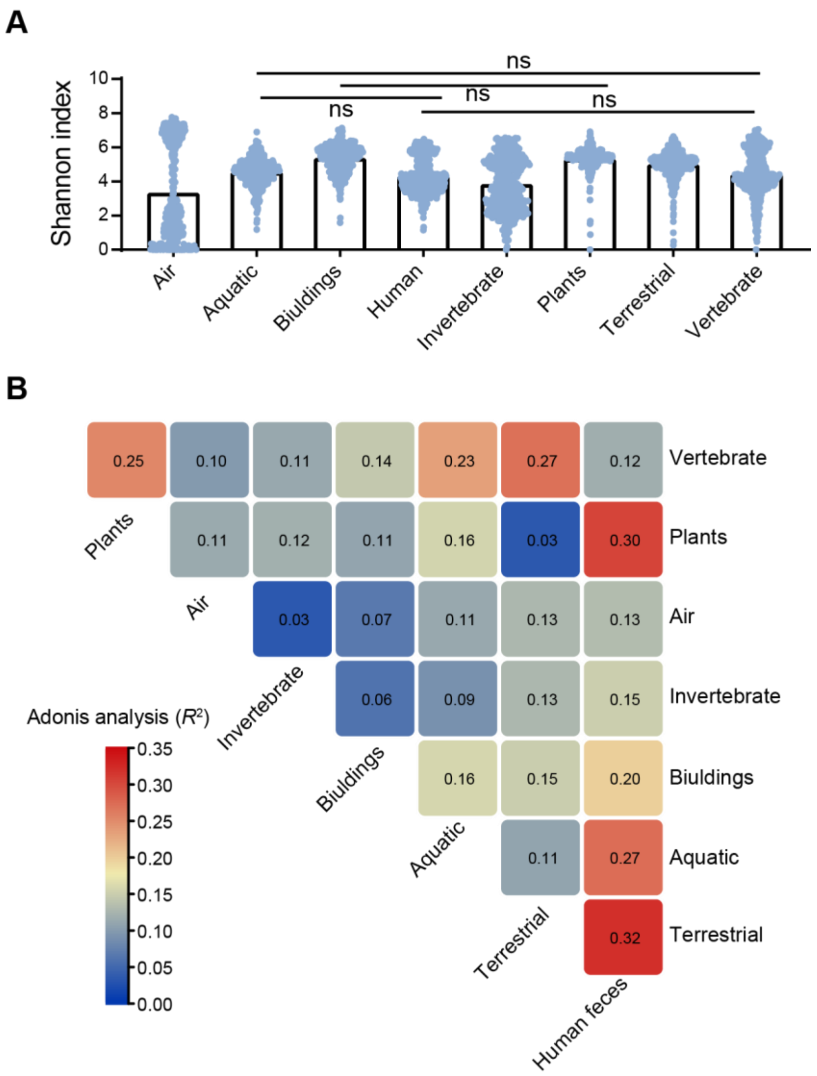

Figure S8. The diversity of resistome in different habitats. (**A**) The Shannon index of resistome from various habitats. "ns" indicates no significant difference between the groups (Kruskal-Wallis test). (**B**) Adonis analysis based on the resistome from different habitats. Color gradation shows the R^2 values (high: red, blue: low).

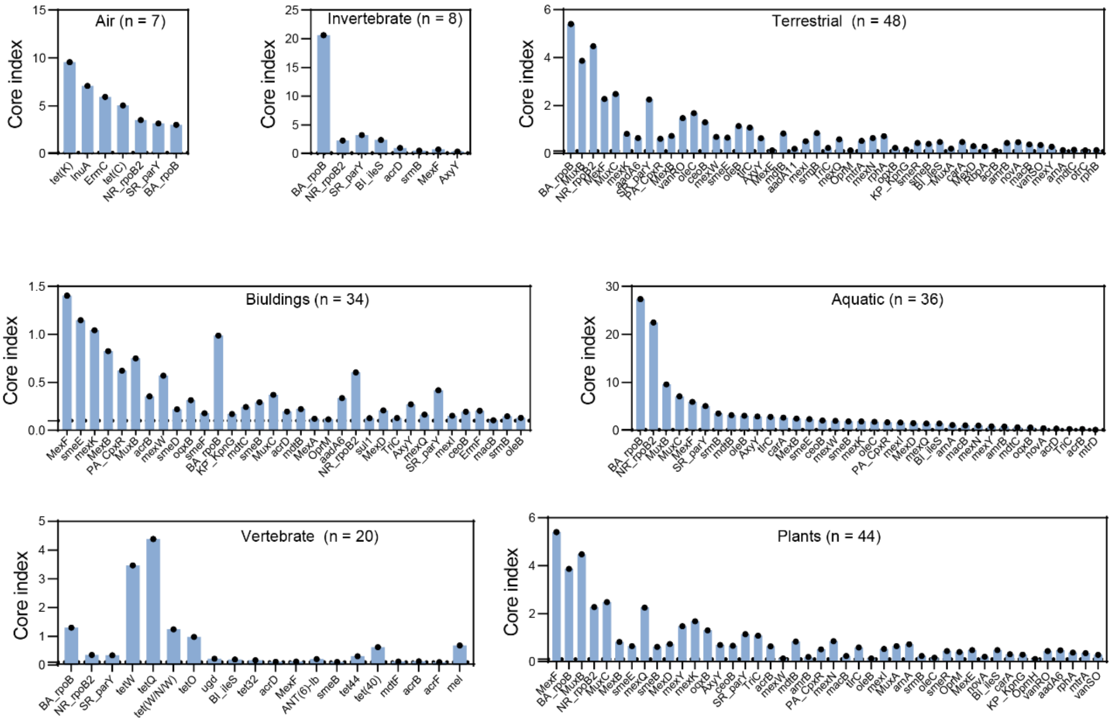

Figure S9. Core ARGs of various habitats. We identified 7, 36, 34, 44, 8, 48 and 20 core ARGs from the air, aquatic, building, plant, invertebrate, terrestrial and vertebrate microbiomes, based on the threshold value (see "Method" section, CI > 0.1 and frequency of studies >60%).

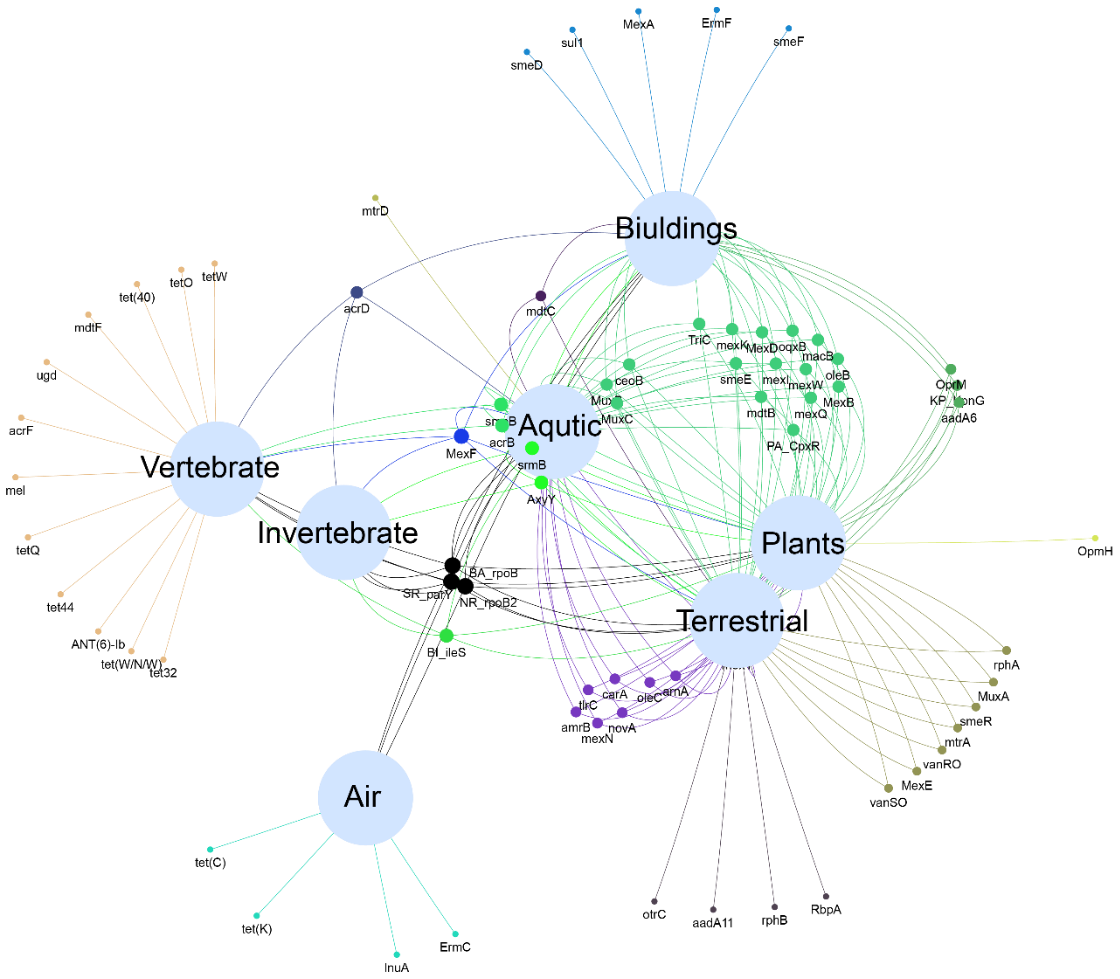

Figure S10. Shared and unique core ARGs among various habitats. The shared network of core ARGs from different habitats presented a similar habitat specificity with the structure of the resistome.

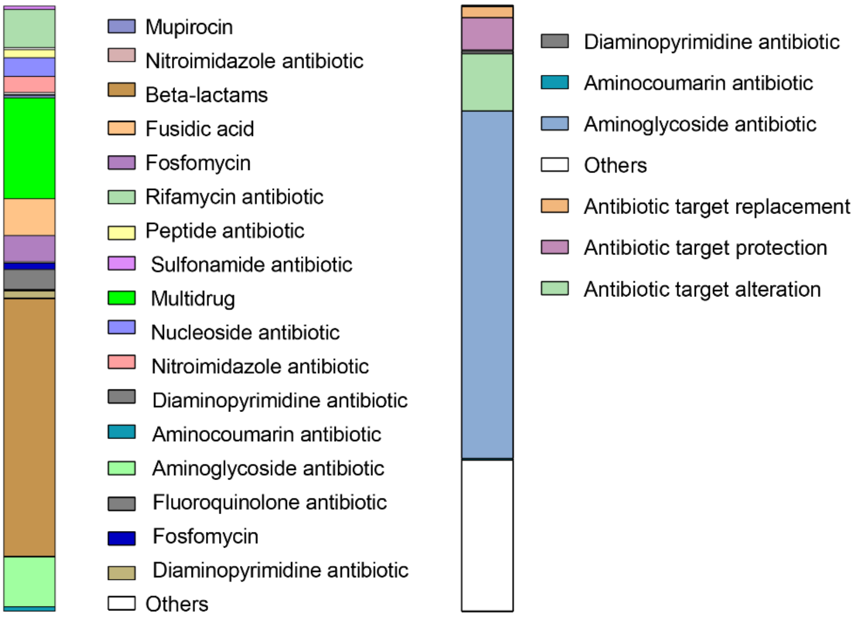

Figure S11. Composition and mechanism of resistance of the shared antibiotic resistome. About 28% of the ARGs were shared across human feces and the other habitats, which mainly conferred resistance to beta-lactams and multidrug and performed most of the inactivation and efflux of antibiotics.

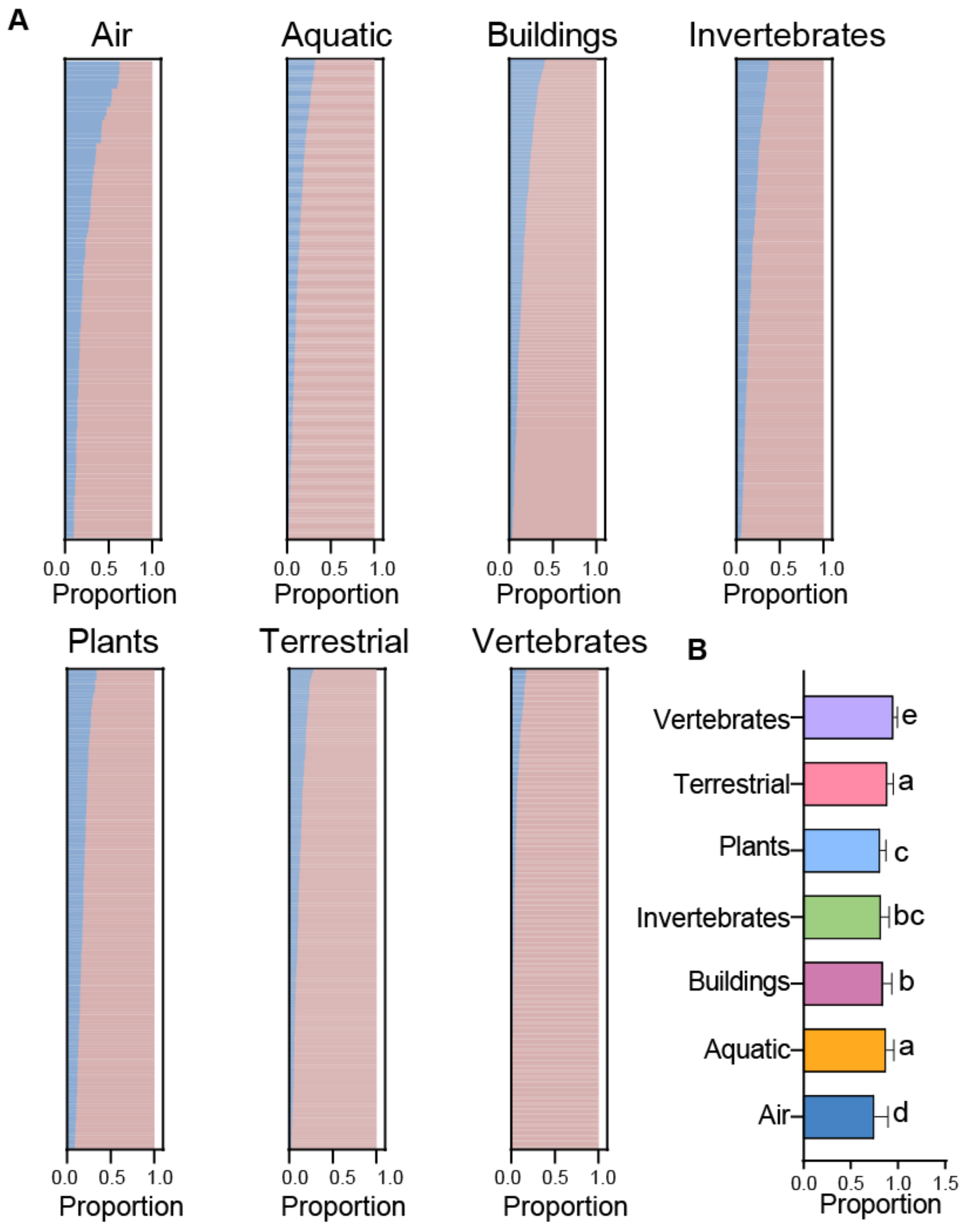

Figure S12. Source proportion of human fecal resistome to the different habitats. (**A**) Fast expectation-maximization for microbial source tracking (FEAST) estimating the source contribution of human fecal resistome to the different habitats. (**B**) Vertebrates harbour more human feces-derived resistome among all habitats. Different letters represent significant differences between habitats (Kruskal-Wallis test, adjust $p < 0.05$).

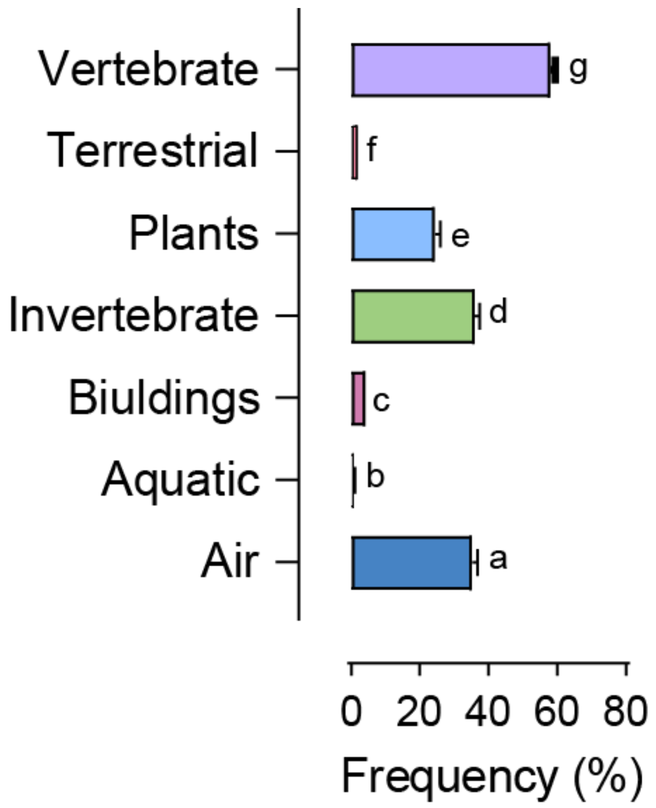

Figure S13. Frequency of human fecal ARG hosts in various habitats. Different colour dots represent the Shannon index of various habitats and human fecal samples. ns, not significant (Kruskal-Wallis test, adjust $p < 0.05$).

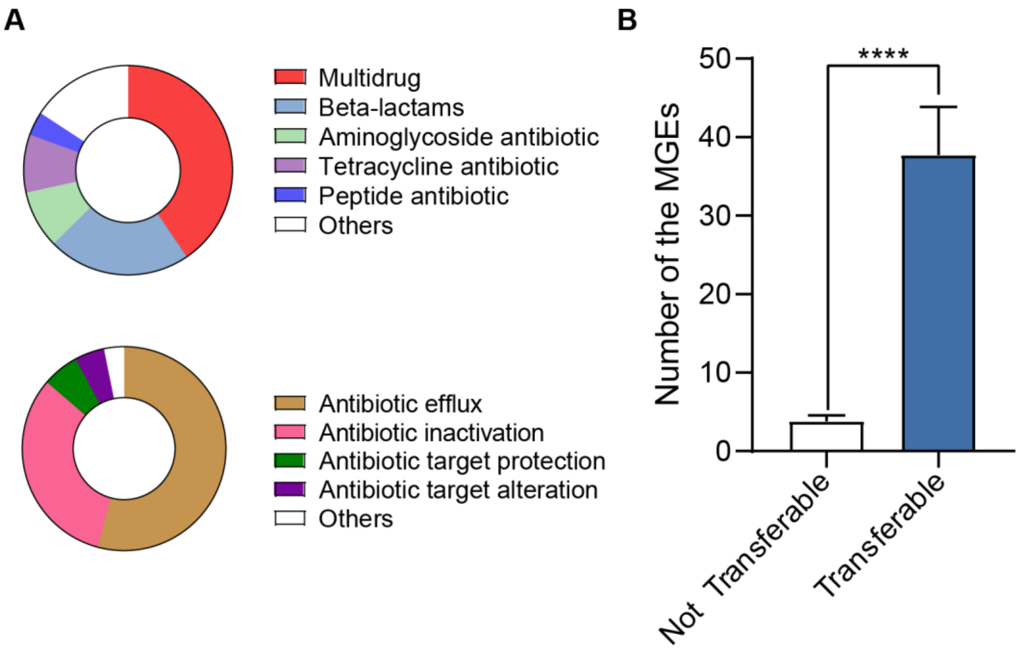

Figure S14. Information for transferable ARGs. (**A**) Classification and mechanism of resistance of transferable ARGs. (**B**) Number of MGE types of transferable and non-transferable ARGs (both their upstream and downstream 5-kb flanking regions in each bacterial genome). ****, adjust $p < 0.0001$ (Mann-Whitney test).

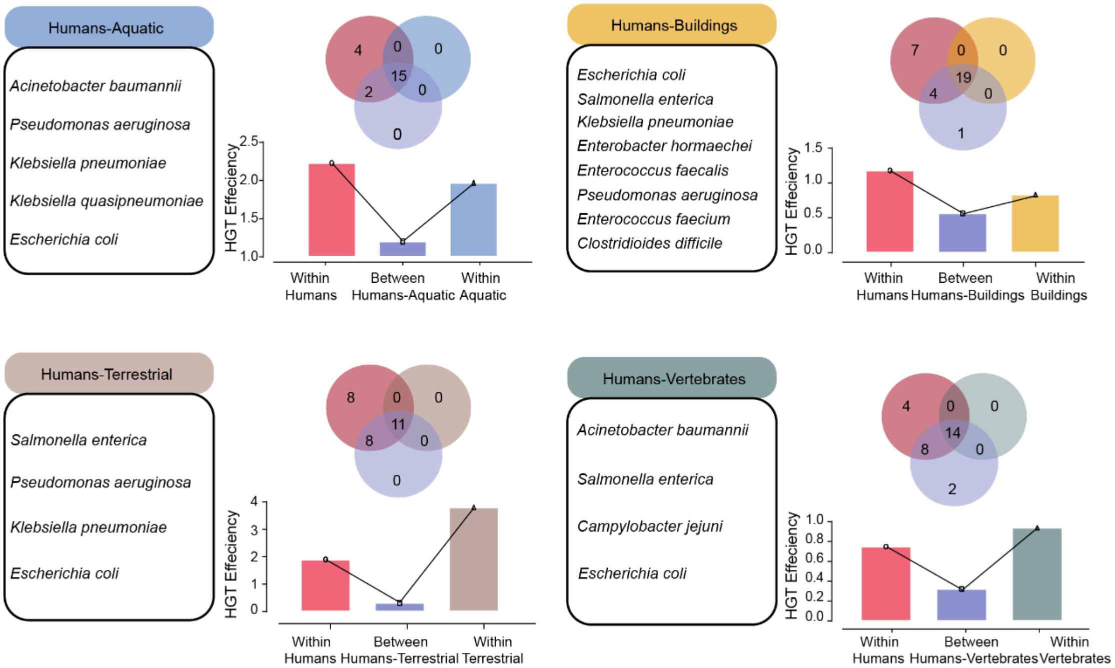

Figure S15. HGT efficiency of ARGs between species in various transmission routes (aquatic-human, terrestrial-human, building-human and vertebrate-human). HGT efficiency between species was significantly higher in the same habitats than in different habitats; terrestrial and vertebrate habitats had higher efficiencies of transfer between species than human feces. The left box is the name of the genome selected, and the Venn diagram shows the number of transferred genomes between species across or within habitats (upper right).

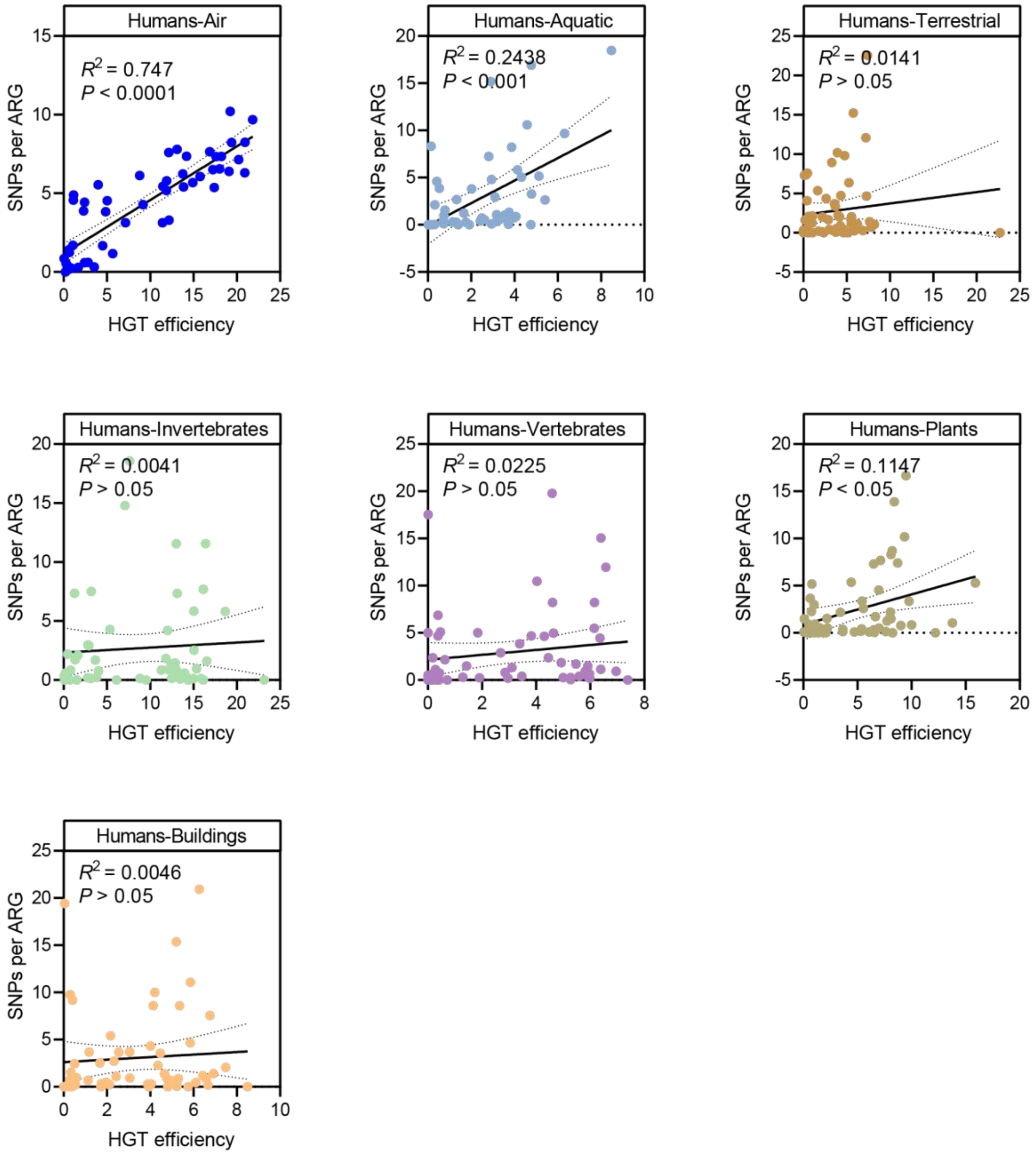

Figure S16. Relationship between the average number of SNPs and the efficiency of transfer of each transferable ARG from the various routes of cross-transmission. The number of SNPs was positively correlated with the efficiency of transfer of each ARG across various routes of cross-transmission (OLS linear regression analysis).

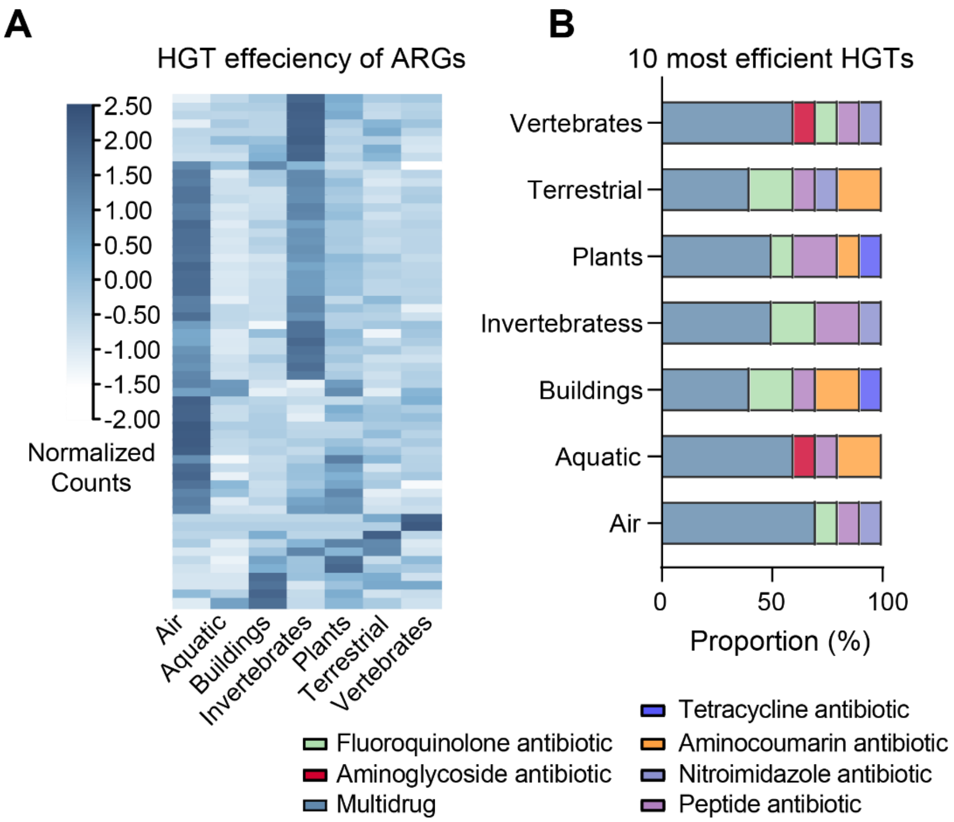

Figure S17. Efficiency of the horizontal transfer of ARGs in various transmissions across habitats. (**A**) Heatmap of the efficiency of transfer of transferable ARGs within *E. coli* strains in various routes of transmission. Colour gradation indicates the normalized counts of ARGs. (**B**) The classification of transferable ARGs (10 most efficient HGTs) in various routes of transmission.

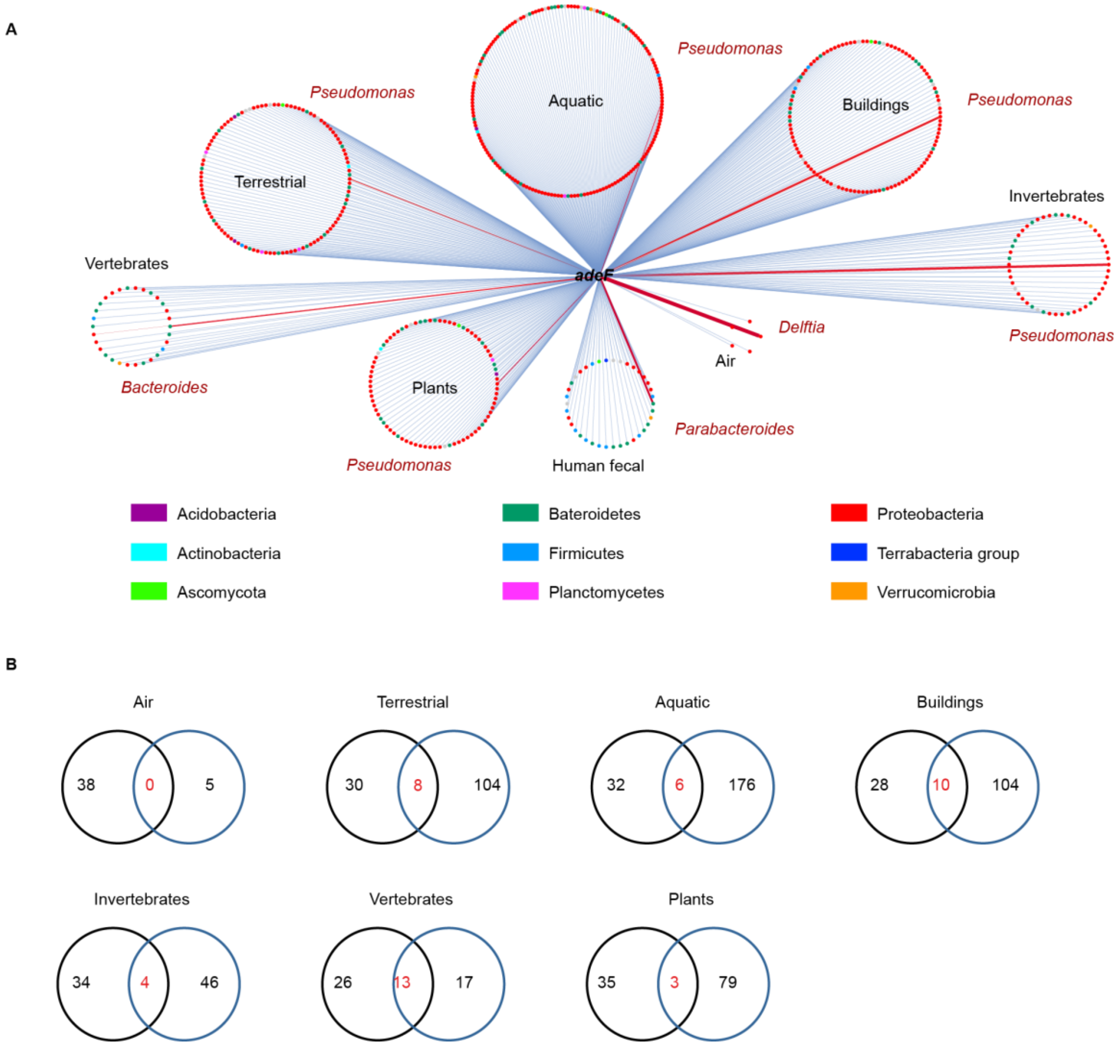

Figure S18. Differences in ARG hosts across human feces and various other habitats. (**A**) The bacterial hosts of *adeF* varied considerably across human feces and other habitats. Different colour of the circle indicates the taxonomy of the ARG host (at the phylum level). (**B**) The bacterial hosts of *adeF* from human feces were rarely shared with the other habitats, implying the filtering of habitats for the ARG hosts.
